# Achievement of 15-Minute Adaptive PCR Benchmark with 1370 nm Laser Heating

**DOI:** 10.3390/bios15040258

**Published:** 2025-04-17

**Authors:** Nicholas Spurlock, Rosana Alfaro, William E. Gabella, Kunal Chugh, Megan E. Pask, Franz Baudenbacher, Frederick R. Haselton

**Affiliations:** 1 Department of Biomedical Engineering, Vanderbilt University, Nashville, TN 37235, USA; nicholas.a.spurlock@vanderbilt.edu (N.S.); kunal.chugh@vanderbilt.edu (K.C.);; 2 Department of Physics and Astronomy, Vanderbilt University, Nashville, TN 37235, USA; bill.gabella@gmail.com

**Keywords:** point of care, molecular diagnostics, rapid PCR, photothermal heating, adaptive PCR, plasmonics

## Abstract

In low-resource and point-of-care settings, traditional PCR often faces challenges of poor sample preparation, adverse environmental conditions, and long assay times. We have previously described a laboratory-based instrument to achieve “adaptive” PCR, a PCR thermocycling control system that replaces preset cycling times and temperatures with the optical monitoring of added L-DNA stereoisomers matching the sequences of the reaction primers and target. These L-DNA biosensors directly monitor DNA hybridization, compensating for ambient environmental conditions and poor sample preparation. This report describes instrument simplifications and a comparative evaluation of both direct photothermal and plasmonic laser heating to reduce the assay time to 15 min. Instrument performance was assessed using a split sample design to compare reaction performances of 1370 and 808 nm adaptive PCR heating modalities to a standard PCR instrument. Both the novel 1370 nm direct heating and the 808 nm plasmonic method achieved target amplification similar to the traditional PCR system within 15 min. However, a major disadvantage of 808 nm heating was nanorod optical interference that reduced the fluorescence signal from PCR probes and optical cycling components. Further characterization of the 1370 nm direct heating method found comparable limits of detection of 10^0^ copies/µL and reaction efficiencies of approximately 2 for both the 1370 nm system and the traditional PCR instrument. These results suggest that a field-deployable PCR instrument design incorporating both adaptive optical control and 1370 nm laser heating can achieve 15 min sample assay times without sacrificing analytical sensitivity.

## 1. Introduction

Polymerase chain reaction (PCR) uses thermal cycling to change the hybridization state of double-stranded DNA, causing the strands to separate from each other and anneal to short primer sequences, thus enabling replication via primer extension by DNA polymerase. Traditional PCR instrumentation employs preset temperatures to achieve the desired replication while preventing undesirable outcomes such as nonspecific binding [1,2]. However, cycling control based on preset temperatures requires calibration and can be vulnerable to external environmental conditions and changes in primer–target hybridization due to impurities such as salts and alcohols [3,4]. In previous reports, we established optically labeled L-DNA stereoisomers as a surrogate for the molecular interactions of the D-DNA reaction components to achieve cycling control. Rather than using preset temperatures, this “adaptive” PCR approach monitors the molecular interactions of fluorescently end-labeled L-DNA analogs of the D-DNA primer and target sequences. These enantiomeric analogs of the D-DNA sequences do not interact with PCR enzymes or the D-DNA present in the reaction. Importantly, these biosensors accurately mirror the annealing and melting characteristics of their D-DNA counterparts [5,6], and thus their optical measurements also reflect the influence of factors that affect hybridization, such as changes in temperature and sample impurities like salts and alcohols. This cycle control method, which replaces instrument calibration, has been experimentally verified to adapt to environments that cause traditional PCR instruments to fail [7] and, in some cases, achieved PCR detection without extraction or other sample preparation steps [8,9]. These properties are ideal for low-resource and point-of-care field settings [10], where temperature-controlled laboratories and extensive operator training are uncommon.

A major design goal in these settings is a 15 min assay time. Like most commercial PCR instruments, the previous laboratory-based instrument required 45 to 60 min for a full 40-cycle PCR reaction. Reducing the assay time is an important goal in any setting, as reducing the assay time leads to increased throughput, but it is particularly important in low resource and point-of-care settings where a shorter assay time is important to optimize clinical decision making within a single visit. A 15 min assay time is desirable in these scenarios, and most lateral flow assays and other simplified tests return a result within this time frame [11]. Others have shown that reaching a 15 min PCR assay time is possible, but methods to do so are often difficult to implement in point-of-care settings, requiring, for example, custom fabricated chips [12,13] or small, difficult to handle volumes [14,15].

Like many others, we focus on decreasing the heating and cooling cycling time to reduce the time-to-result [16,17,18,19,20,21]. Increasing the cooling or heating ramp rate is one means to shorten the time it takes to reach the optimum reaction temperatures [19], and, of the two, increasing the heat ramp rate is the simplest to implement. Our previous implementation of the laboratory-based adaptive instrument relied on hot air to heat by thermal conduction through the plastic wall of the sample tube. However, simply increasing the temperature on the outside of the sample tube to increase the ramp rate risks melting or otherwise deforming the tube wall. Therefore, using hot air to heat is not compatible with the goal of reaching a 15 min assay time. One potential method of increasing the heat ramp rate without affecting the sample tube wall is photothermal heating using wavelengths not absorbed by the sample tube wall. Plasmonic PCR methods, which heat water indirectly through the use of a gold nanorod intermediary [18,22], and infrared wavelengths that are directly absorbed by water [16,20] are both viable options for increasing the heating rate and potentially reducing adaptive PCR sample assay times to 15 min or less.

This report evaluates both indirect heating with an 808 nm laser and gold nanorods and direct heating with a 1370 nm laser to achieve a 15 min assay time. We also describe the incorporation and evaluation of these new heating methods with a new, self-contained and open-source instrument architecture that reduces costs and external equipment as a step toward a field-deployable design of “adaptive” PCR with the goal of achieving an assay time of 15 min with the well-established PCR reagent supply chain and workflow.

## 2. Methods

**Figure 1:**
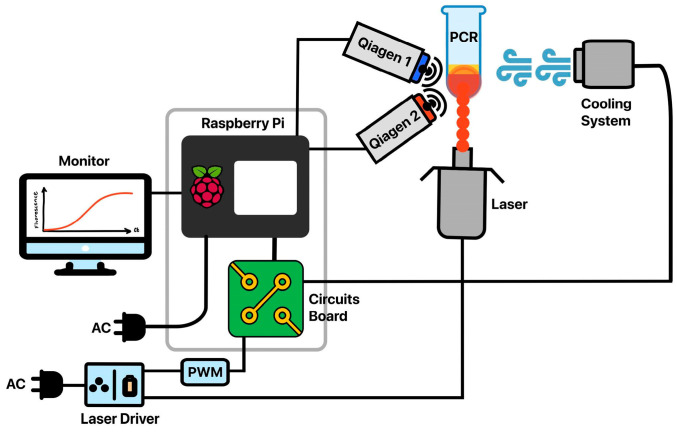
Hardware diagram of the photothermal PCR instrument. The cooling system and laser are both routed through relays on the circuit board, with pulse width modulation implemented for control of laser power in the 808 nm version of the device. The 1370 nm instrument controls the laser driver through a digital-to-analog converter chip instead. The optics (two Qiagen ESELOG detectors) are connected to the Raspberry Pi via USB and used to inform cycling decisions and for real-time PCR data collection. The ADCs and DAC chip are both part of the circuit board and are not pictured separately.

**Figure 2:**
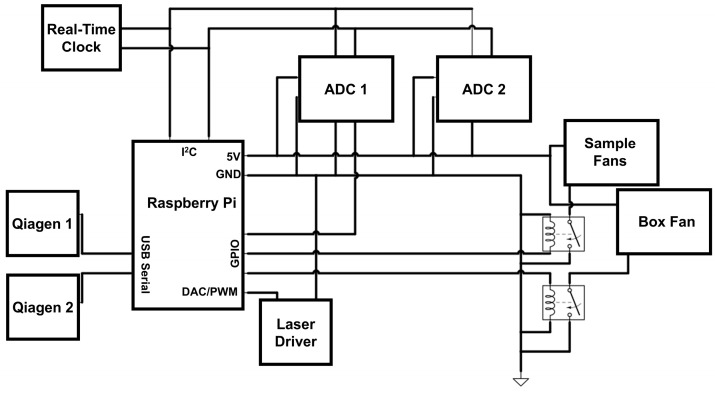
Circuit diagram of the 808 nm L-DNA-controlled instrument. Relays and laser driver use the GPIO pins from the Raspberry Pi, which also provides power to the fans and ADCs for the thermocouples. Communication with the ADCs is through the I^2^C bus, while the detectors use a USB serial connection. The 1370 nm instrument is similar, but an I^2^C digital-to-analog converter replaces the PWM interface.

Instrument Design and Construction. Many aspects of the instrument’s hardware design were carried over from our previously published instrument. As in our previously published design [7], optical detection used two dual-channel Qiagen ESElog USB confocal fluorescence detectors (DIALUNOX, Stockach, Germany). The optics were focused on a 20 μL reaction volume contained within a polypropylene 0.2 mL PCR tube (ThermoFisher Scientific #AB-0620, Waltham, MA, USA). The orange (565 nm excitation, 625 nm detection) and yellow (520 nm excitation, 570 nm detection) channels were used to collect the fluorescence data required for anneal and melt control, respectively, while the green channel (470 nm excitation, 520 nm detection) was used to detect the real-time PCR. The red channel (625 excitation, 680 detection) was unused. The Qiagen detectors used USB serial connections, separate from the rest of the control circuitry. Also, like the previous design, two cooling fans (Product # 179-CB5015BF05539120, Mouser Electronics, Mansfield, TX, USA) were powered and controlled via relays (G6DN-1A, DigiKey, Thief River Falls, MN, USA) connected to the control circuitry. These fans were focused on the sample with 3D printed air ducts (STL files for all 3D printed parts are available upon request). To remove the original instrument’s requirement for external devices and proprietary software, a Raspberry Pi 4B (Cana Kit Corporation, Vancouver, BC, Canada) was used to control and manage the instrument (Figure 1). The Raspberry Pi was housed in the instrument and connected to the other components via a ribbon cable and printed circuit board (JLCPCB, Zhuhai, China), as shown in Figure 2. (EAGLE Files for the printed circuit board are available upon request). K-type thermocouples were attached to the instrument through two I^2^C ADS1115 analog-to-digital converters (ADCs, Product #1085, Adafruit, New York City, NY, USA) to provide instrument thermal data if needed. An I^2^C real-time clock chip (DS3231 Adafruit, New York City, NY, USA) was used to ensure reliable timestamps for fluorescence and temperature data. Power to low-voltage components was supplied by the Pi’s onboard 5V supply.

The functionality of the previous L-DNA-controlled PCR software (Version B.3.2) was maintained in a new software architecture consisting of 3 primary modules, a low-level C++ hardware management “engine”, a Node.js server, and an HTML/JavaScript interface. The C++ “engine” coordinates PCR control and data collection by interacting with the hardware components. This engine includes the cycling control algorithm, which operates on the same principles as the previous instrument’s version [7], using two double stranded L-DNA analog sequences: a melt sensor sequence and an annealing sensor sequence (Table 1). These sequences hybridize and melt at the same temperatures as the long target sequence and shorter primer sequences, respectively, and are each end-labeled with a fluorophore and a quencher. During heating, the fluorescence of the end-labeled melt sensor sequences is monitored, while during cooling the fluorescence of the end-labeled anneal sensor sequences is monitored. In both cases, a Gaussian curve is fitted to the derivative of the fluorescence values and used to predict when the derivative approaches zero—in other words, when the fluorescence stops increasing during heating, or decreasing during cooling. At that time, the system chooses a “switch point” and switches from heating to cooling, or takes a PCR reading and switches from cooling to heating, respectively. Using the derivative makes the system resilient against conditions that might cause the overall magnitude of the fluorescence signal to change. A Node.js WebSocket server manages the interaction between the C++ hardware code and the HTML user interface, transmitting data for visual display to the interface and passing changes in assay parameters from the user interface to the hardware code. HTML was chosen to implement the interface to take advantage of general familiarity with websites and web browsers, as well as the wide range of devices that can display HTML code. This custom instrument management software (Version C.2.1) replaces the LabVIEW interface that was used in the previous version of the instrument. An external monitor, mouse and keyboard were used for monitoring and control in this report, but the instrument can also be controlled wirelessly with any internet-capable device by connecting to a local Wi-Fi network broadcast by the Raspberry Pi.

Two new heating designs were implemented through two different laser assemblies to replace the previous hot air-based design. The 808 design used a preassembled, all-in-one 808 nm laser package that included a power supply and built-in diode cooling fan (Sunshine-Electronics, Wuhan, China) for plasmonic heating. The laser state and power were controlled by a pulse width modulated (PWM) signal from the Raspberry Pi, connected to the laser driver board via a pinout on the printed circuit board. Output power for the laser was measured at 0.93 W. The 1370 nm laser system was assembled in-house with a 2 W 1370 nm laser diode (Thorlabs L1370G1, Newton, NJ, USA) and powered with a constant current source (PLD10K-CH, Edmund Optics, Barrington, ID, USA) at an output power of 1.42 W. Power levels for both lasers were measured by focusing the beam on a Thorlabs PM200 power meter (Thorlabs, Newton, NJ, USA) using the collimating lens. As the aim of this study was to lower the assay time, the highest available power for each laser setup was used. Cooling for the 1370 nm laser diode was accomplished with a machined aluminum heat sink and 5 V fans. The current controller for the 1370 diode was incompatible with the onboard PWM system for the Raspberry Pi, so a digital-to-analog converter chip was used to control the output power of the diode (MCP4728, Adafruit, Brooklyn, NY, USA). For the 808 nm design, a short-pass filter (Edmund Optics, Barrington, ID, USA) with a cutoff wavelength below 800 nm was placed on the optical detectors to prevent optical interference from the laser during heating. The 1370 nm laser did not cause any optical interference with the detector, so the filter was not used with those experiments.

For both lasers, an infrared camera was used for coarse alignment with the PCR volume (Figure S2). Perfect alignment is not critical for the L-DNA-controlled system because the fluorescence control mechanism will compensate for slower heating rates, but the best possible alignment is preferred for maximum power delivery to the reaction volume and thus faster heating. For precise alignment, a paper silhouette of the reaction volume replaced the sample tube and a power meter (Thorlabs PM200, Newton, NJ, USA) was placed behind the silhouette. The laser was carefully moved with optics mounts to maximize power read by the meter. Each laser was aligned once during the assembly phase. For both wavelengths, a collimating lens was used to focus the laser on the reaction volume and tune the spot size to be slightly larger than the sample cross-section to minimize the formation of hot spots and achieve more uniform heating within the reaction volume. In the case of the 808 nm laser, this collimating lens was part of the preassembled kit, while the collimating lens for the 1370 nm laser (ACL12708U, Thorlabs, Newton, NJ, USA) was purchased separately and held by an optical mount.

Oligonucleotides, primers, probes, and nanorods. *S. mitis* target, primers, and probes were synthesized by Integrated DNA Technologies (Coralville, IA, USA). *S. mitis* was chosen for PCR testing from the reagents on hand due to its high analytical sensitivity [23]. Labeled L-DNA oligonucleotides and primers were synthesized by Biomers.net (Ulm, Germany) (Table 1). To achieve the necessary cycling parameters, the L-DNA sensor sequences were selected to have melting temperatures (T_m_) comparable to those of the D-DNA primers and targets. Theoretical melt temperatures were used due to the difficulty of experimentally finding the melt temperatures of short sequences such as primers. These theoretical melt temperatures were calculated using OligoCalc [24], with the salt-adjusted temperatures used for comparison since Luna Universal Probe qPCR Master Mix (New England Biolabs Catalog #M3004L, Ipswich, MA, USA) contains concentrations of various salts. While the specific salt concentrations are proprietary, these approximations are sufficient for comparative purposes. The L-DNA anneal sensor sequence used was a 22 base pair sequence with a T_m_ of 58.4 °C, comparable to the *S. mitis* forward primer melting temperature of 59.4 °C. The melt sensor sequence was a 77 base pair sequence with a melt temperature of 90.8 °C, high enough to ensure denaturing of the double-stranded target *S. mitis* sequence, which had a T_m_ of 89.1 °C (Table 1). Gold nanorods (PCR-808-50-1, Nanopartz Inc, Loveland, CO, USA) were purchased with a proprietary PCR-grade coating to prevent reaction components from interacting with the nanorod surface [18,25].

Cycle quantification and efficiency calculation methods. The performance of both photothermal PCR instruments were compared with traditional PCR by comparing cycle quantification (Cq) values and single-sample efficiencies with a commercial Rotor-Gene Q instrument (QIAGEN Catalog # 9001580, Hilden, Germany). To compare across different PCR systems, all fluorescence measurements were normalized to a 0 to 100 relative fluorescence scale by subtracting the baseline, dividing by the highest fluorescence measured, and multiplying by 100. Baselines were calculated by averaging the PCR fluorescence across the first 15 cycles. Quantification cycle (Cq) value was calculated as the first value where measured fluorescence was 10 standard deviations above the baseline. A spline interpolation was used to estimate fractional Cq results. Single-sample PCR efficiencies in the absence of a standard curve were calculated with a method similar to that established for LinRegPCR [26]. The logarithm of the normalized fluorescence was calculated for each sample, and a window of linearity selected that corresponded to the exponential phase of the reaction. The slope of the linear fit for this window of linearity is equivalent to the natural logarithm of the efficiency of the reaction. For consistency, the window of linearity was set to be 5 PCR fluorescence measurements, centered on the nearest cycle number to the fractional Cq value.

808 nm PCR system performance. The performance of the 808 nm system was evaluated via Cq and single-sample efficiency comparisons with paired samples in a traditional Rotor-Gene PCR instrument. All samples were performed in 1X Luna master mix (Luna Universal Probe qPCR Master Mix, New England Biolabs Catalog #M3004L), and prepared according to the manufacturer instructions in standard 0.2 mL PCR tubes (ThermoFisher Scientific #AB-0620, Waltham, MA, USA). Forward and reverse primers were added at a concentration of 400 nM each, while the hydrolysis probe was added at a concentration of 200 nM (all sequences are listed in Table 1). The synthetic *S. mitis* target was added at a concentration of 10^7^ copies/µL for a final sample concentration of 10^6^ copies/µL. L-DNA anneal and melt sensor sequences were added at concentrations of 125 nM and 312 nM for the fluorescing and quenching sequences for each, respectively. Samples were prepared with nanorod concentrations of 3.15 nM, 6.3 nM, and 9.45 nM (n = 6 per concentration), and molecular grade water was added to bring the samples to a final volume of 20 µL. All samples were covered with 15 µL of PCR grade mineral oil (Sigma-Aldrich #M8662, St. Louis, MO, USA) to prevent evaporation during heating and the samples were placed in the L-DNA-controlled PCR instrument and set to run for 40 PCR cycles. As in our prior work, no hot start was used for the L-DNA instrument [7]. PCR of paired samples at each nanorod concentration (n = 2 per concentration) were also performed in the Rotor-Gene Q instrument and the Cq results were compared. For both instruments, an additional non-template control (NTC) group was performed with a 6.3 nM nanorod concentration. The thermocycling of the Rotor-Gene Q had an initial hold at 95 °C for 3 min, then 40 repeating cycles of 95 °C for 15 s, and 60 °C for 60 s. Average cycle time for the L-DNA-controlled instrument was calculated by averaging the cycle time over 10 cycles, from cycle 2 to cycle 11. Excluding the first cycle ensures any time variation due to a different starting or environmental temperature is also excluded. Since results with 6.3 nM were consistent in the concentration experiments, additional 6.3 nM samples were prepared as above and performed in the adaptive PCR to measure the variance in the PCR performance in achieving positive results in a 15 min timeframe (n = 13).

Gold nanorod optical interference experiments. FAM, HEX, and Texas Red fluorophores were used to measure the optical interference of the nanorods in different fluorescence channels. Single stranded DNA functionalized with FAM, HEX, and Texas Red dyes were added at a final concentration of 75 nM each to Luna master mix. Nanorods were added at final concentrations of 0 nM, 3.15 nM, 6.3 nM, and 9.45 nM for each sample group, and molecular grade water was added to bring the total reaction volume to 20 µL (n = 3). The fluorescence detectors on the L-DNA-controlled instrument were used to collect raw fluorescence data, starting at the lowest possible power on each channel, and gradually increasing power delivered to the excitation LED while measuring accompanying fluorescence. Fluorescence blocking was a constant factor across all gains, so the fluorescence at each gain was divided by the corresponding fluorescence of the control sample to find the fraction of light remaining, then those fractions were averaged across the entire gain response curve for the final measurement.

1370 nm PCR system performance. PCR efficiency of the 1370 nm system was compared to the commercial PCR instrument via both the Cq value and single-sample efficiency described above, and a further quantitative PCR standard curve for a traditional comparison of efficiency as well as a limit of detection study. Samples were prepared in standard 0.2 mL PCR tubes with Luna Universal Probe qPCR Master Mix according to manufacturer instructions. *S. mitis* PCR reaction components were added at the same concentrations as the 808 nm experiments, with both forward and reverse primers added at 400 nM and the probe at 200 nM. During the preliminary experiments for these reactions, it was observed that the low concentrations of the *S. mitis* target used in the standard curve interacted with the tube walls during serial dilution, so 1 µM (dT)_21_ (sequences consisting of 21 consecutive thymine bases) was added to each serial dilution to mitigate these potential binding events [27]. The same L-DNA sequences were used for these tests (Table 1), at 100 nM for the fluorescing anneal and melt sensor sequences, and 300 nM for the respective quenching sequences. Serial dilutions of the synthetic *S. mitis* target were prepared at decreasing concentrations from 10^6^ to 10^0^ copies/µL. These were added to the reaction volume for final concentrations of 10^5^ to 10^−1^ copies/µL in each 20 µL sample, and paired identical samples (n = 4 per concentration) were placed in the Rotor-Gene Q and L-DNA-controlled instrument for 40 PCR cycles. Like the samples for the 808 nm comparison, the Rotor-Gene Q was set to run for an initial hold at 95 °C for 3 min, then 40 repeating cycles of 95 °C for 15 s, and 60 °C for 60 s. The 1370 nm samples had a layer of 10 µL of mineral oil to prevent evaporation. As in our prior work and the 808 nm system, no hot start was used for the 1370 nm heating method [7]. All concentrations were used in the formation of the standard curve, while the 10^5^ copies/µL samples were used for the direct comparisons of Cq and single-sample efficiency to mirror the 808 nm methodology. Results were analyzed for Cq value using the method described above. Quantification time was calculated using the timestamps associated with PCR readings in place of cycles.

The data for the standard curve samples were collected while taking PCR measurements on all four fluorescence channels, which provides more testing data but slows cycling due to LED rise times. Therefore, additional samples were performed to identify the minimum assay time possible with the 1370 system architecture, using 10^5^ copies/µL samples like those used in the standard curve above (n = 4, NTCs run in triplicate). For these samples, only the green channel was used for real time PCR reading, rather than multiplexing with all four channels.

Variation in the heating and cooling processes of the 1370 nm instrument architecture were also examined using cycles 2–16 from the samples performed for the standard curve. The fluorescence control curves from the L-DNA end-labeled sequences were extracted and evaluated. The fluorescence measurements at the anneal and melt switch points for cycles 2 through 16 were averaged and the deviations for each individual cycle from the sample’s average anneal and melt switch points were calculated. Variation in the heating and cooling times were calculated the same way, from the same data, and the corresponding data were collected and calculated from the 31 6.3 nM samples on the 808 nm instrument for comparison. Cycles 2 through 16 were chosen as they exclude both the fluorescence from the PCR TaqMan probe, and any time variation in the first heating cycle due to different environmental starting temperatures. To characterize the waste heat of the 1370 nm system, a thermocouple was attached to the laser heat sink for an entire sample run to measure overall heat generated by the 1370 nm laser’s operation. Temperature ramp rate for the system was estimated by placing a thermocouple directly in a reaction volume that only contained L-DNA control sequences and Luna Universal Probe qPCR Master Mix, replacing the volume of PCR reagents with water. The system was set to run for 20 “PCR” cycles, and the average heating and cooling rates estimated from the ramp rate measured over these 20 cycles. While this method is potentially vulnerable to interference from the laser heating the thermocouple directly, the L-DNA control mechanism will reflect true changes in molecular interactions.

## 3. Results and Discussion

**Figure 3:**
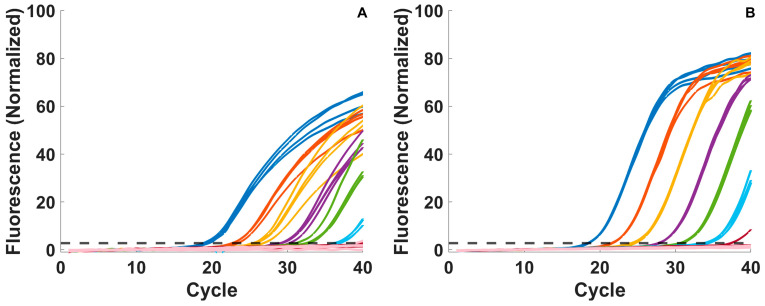
(**A**) Standard curves for adaptive PCR with 1370 nm heating and (**B**) corresponding commercial Rotor-Gene PCR curves. Concentrations are 10^5^ copies/µL (dark blue), 10^4^ copies/µL (red), 10^3^ copies/µL (yellow), 10^2^ copies/µL (purple), 10^1^ copies/µL (green), 10^0^ copies/µL (light blue), and 10^−1^ copies/µL (magenta). NTCs are indicated by the light pink lines.

The new architecture incorporating L-DNA control of photothermal PCR consistently produced assay results that agreed with commercial PCR instrumentation in a 15 min timeframe for the 1370 nm direct heating experiments and a subset of the 808 nm indirect heating experiments. All samples for the 1370 nm returned positive results with consistent PCR Cq values (Figure 3A) down to 10^0^ copies/µL with comparable outcomes to the commercial PCR instrument (Figure 3B). Across multiple samples at each concentration, Cq standard deviation was less than 1 cycle. More variability was observed in the 1370 nm data, which is to be expected as each of the 32 samples in the 1370 nm system was tested individually, while the Rotor-Gene samples were tested in two batches of 16 each, but the variation is still relatively small. Higher target concentrations generally displayed less variability in Cq than lower concentrations, with the standard deviation ranging from 0.30 at 10^5^ copies/µL to 0.75 at 10^1^ copies/µL, apart from the lowest concentration above the limit of detection (10^0^ copies/µL), which had a standard deviation of 0.31.

**Figure 4:**
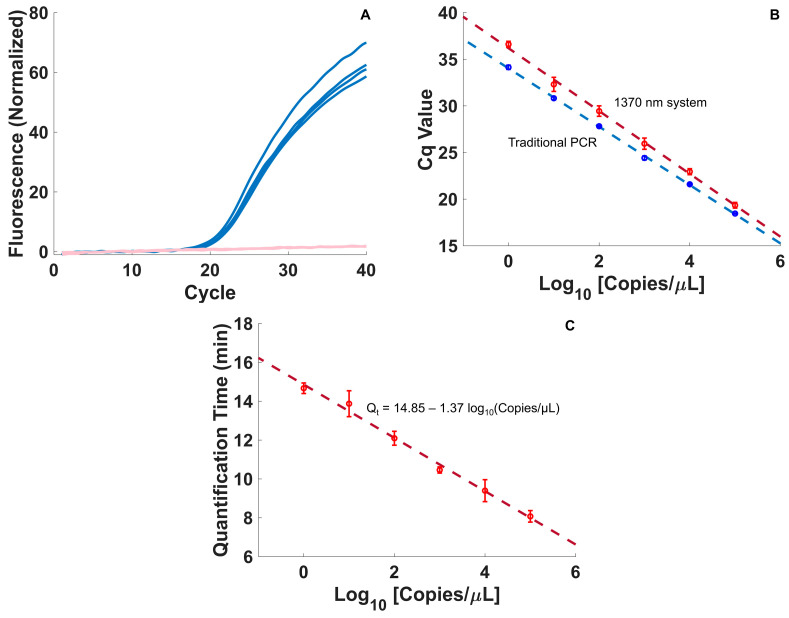
Performance of the 1370 nm direct heating system. (**A**) Singleplex PCR data from the 1370 nm PCR instrument (blue, n = 4) show consistent Cq performance (20.5 ± 0.35) in less than 15 min at a target concentration of 10^5^ copies/µL. Forty cycles were completed in 14.62 ± 0.42 for all seven samples. Triplicate NTCs (pink) returned negative results. (**B**) Standard curve comparison between the 1370 nm system (red) and traditional PCR system (blue) show that the 1370 nm system has comparable performance to traditional PCR instrumentation (n = 4, mean ± SD). (**C**) Quantification time as a function of target concentration (red) shows that a positive result can be obtained within 15 min, with results under 10 min for high copy numbers.

In singleplex fluorescence testing at a target concentration of 10^5^ copies/µL, the 1370 nm PCR system returned consistent PCR results (Cq of 20.5 ± 0.35, n = 4), with a total assay time of 14.62 ± 0.42 min (Figure 4A) and an average cycle time of 21.76 ± 0.64 s. Standard curves in both the traditional PCR instrument and the 1370 nm L-DNA-controlled instrument also produced consistent results for each sample concentration (n = 4 for each system), with similar efficiencies across all target concentrations (Figure 4B). In paired samples at 10^5^ copies/µL, the 1370 nm exhibited comparable PCR performance to the traditional PCR system, with a Cq value of 19.3 vs. 18.3 for the traditional PCR instrumentation and single sample efficiencies of 1.50 vs. 1.60. Standard curve PCR efficiency was similarly comparable, with a value of 1.98 for the 1370 nm system and 2.08 for the traditional instrument.

The small drop in efficiency from the traditional PCR system to the 1370 nm system could potentially be due to the lack of an annealing hold step, but the change in efficiency had little effect on the limit of detection when compared to traditional PCR. In both instruments, the limit of detection was 10^0^ copies/µL, with 10^−1^ copies/µL exhibiting false negatives in 4/4 of the 1370 nm samples and 3/4 of the traditional PCR samples (Figure 3). The Cq values are otherwise consistent in both systems, and the L-DNA-controlled system’s adaptation to changing environmental conditions has other advantages, including resisting external factors that would diminish the commercial system’s performance to the point of failure [7,9], as well as the obvious advantages in terms of speed—the average total time for a 40-cycle PCR assay among the 32 samples used for these comparative tests was 16.42 ± 0.69 min with a cycle time of 24.5 ± 1.16 s, compared to approximately 95 min for 40 cycles on the commercial instrument. Crude estimates for heating and cooling rates made with an immersed thermocouple were 2.41°C/s and 2.49 °C/s, respectively. Moreover, the standard curve data using the 1370 nm heating method show that even in the fully multiplexed mode, all positive samples down to the limit of detection were identified before the 15 min mark, with a consistent, linear relationship decreasing the time to detection with the log of copies/µL (Figure 4C).

**Figure 5:**
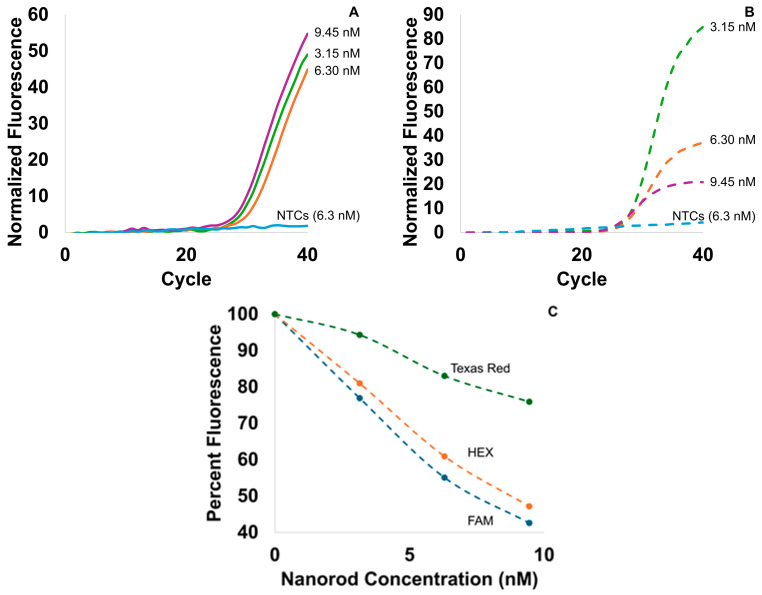
Performance of the 808 nm indirect heating system. (**A**) Averaged 808 nm PCR results for three different nanorod concentrations (n = 6). The plot for 9.45 nM reflects the single positive result (1/6). (**B**) Average Rotor-Gene PCR controls for the 808 nm samples (n = 2) had similar shapes but reduced fluorescence values as the nanorod concentration increased. (**C**) The percent fluorescence of samples containing nanorods relative to 0 nM control samples shows that the nanorod concentration interfered with fluorescence measurements on all three fluorescence channels used in these experiments (n = 3).

The 808 nm system returned true positives for all samples at nanorod concentrations up to 6.3 nM (Figure 5A), but the Cq exhibited greater variability and variability was increased by higher nanorod concentrations. The optimal nanorod concentration for L-DNA-controlled plasmonic PCR was found to be 6.3 nM to ensure target cycling speeds were met while avoiding fluorescence signal loss. Nanorod concentration experiments demonstrated little difference in Cq (Figure 5A) but a high variation in cycle time and assay performance. Increasing the concentration of nanorods from 3.15 nM to 6.3 nM decreased PCR cycle time from 24.6 ± 0.59 s to 22.5 ± 0.80 s, but increasing the concentration further slowed cycling and increased variability, with a cycle time of 24.02 ± 2.15 s. All positive samples in the 808 nm instrument returned an average Cq value of 29.9, while traditional PCR experiments averaged a Cq of 27.2 (Figure 5B). The 9.45 nM samples, however, frequently produced control errors and false negatives. Since the 6.3 nM samples produced consistent results while meeting the 15 min benchmark, additional samples to test consistency were performed at that concentration. Overall, 808 nm plasmonic PCR with a nanorod concentration of 6.3 nM had 100% sensitivity for 10^6^ copies/µL samples in repeated experiments (N = 19, Cq = 29.7 ± 1.38). The average cycle time for all 6.3 nM experiments was 22.8 ± 1.07 s. This would lead to a 15.1 min assay time for a 40-cycle PCR reaction, acceptably close to the 15 min target. Like the 1370 nm dilution curve, these samples were performed with multiplexed PCR readings, which suggests a similar improvement in assay time would be achieved if the fluorescence was limited to a single channel.

The presence of nanorod-related interference was clear, and this interference was further characterized on the 808 nm instrument. Since previous research [22] has shown that functionalized nanorods can be present in concentrations of up to 29 nM without PCR interference, the cause of the false negative results at 9.3 nM is more likely due to the control algorithm inconsistency caused by fluorescence blocking rather than interference with the PCR reaction itself. Indirectly, this interference is reflected in differences in the plateau height of the Rotor-Gene experiments (Figure 5B), since the instrument uses a constant fluorescence gain for all samples, and is further indicated in the reduced fluorescence of the direct scans as a function of nanorod concentration (Figure 5C). The greatest reduction in fluorescence signal was seen on the yellow (HEX) and green (FAM) channels, with over half the fluorescence signal lost at a concentration of 9.45 nM. This wavelength-specific effect is likely because the peak absorbance of the transverse plasmon band of the nanorods is at 508 nm, only 12 nm from the instrument’s yellow excitation/green emission filter peak of 520 nm. However, this interference was present to a lesser extent even on the orange channel, which excites at 565 nm. Optical interference is reflected in the 6.3 nM measurements of cycle time (Figure S4B), and the cycle time distribution grew wider with increasing nanorod concentrations, from a standard deviation of 0.59 s at 3.15 nM to 2.15 s at 9.45 nM. Moreover, increasing the nanorods from 6.3 nM to 9.45 nM demonstrated no further increase in cycling speed. Calculating the theoretical absorbance of each nanorod concentration shows that only 3.7% of the light is not absorbed in even the narrowest path through the bulk of the reaction volume at a nanorod concentration of 6.3 nM, while 0.71% escapes in the 9.45 nM samples. The comparatively small increase in power absorbed from 6.3 nM to 9.45 nM could explain the negligible effects on cycling speed—past a concentration that has near total absorbance, additional nanorods will have little effect on heating speed. Differences in absorption also explain the similar cycling times between the 808 nm and 1370 nm systems despite the large difference in the laser power (0.93 W vs. 1.42 W). While most of the light is absorbed in both cases, 17% of the 1370 nm power passes through the thickest part of the sample without being absorbed, while only 1.1% escapes from the nanorod samples (Figure S5A,B). This difference is greatest where the sample thickness is smallest.

The heat delivered within the reaction volume is not uniform, which leads to heterogeneity in the hybridization state of the PCR components. We attempted to minimize lateral heterogeneity—that is, nonuniformity perpendicular to the laser beam—by matching the laser spot size to the sample volume. However, it is likely that there is still heterogeneity in the hybridization state with respect to depth. During heating, the laser heats the reaction volume exclusively from one side, and power absorbed follows an exponential decay according to Beer’s Law. During cooling, the reaction volume is cooled from the outside inward during cooling, suggesting that the temperature—and, by proxy, the molecular interactions—in the core of the sample lags behind that of the exterior, as previously discussed in other rapid PCR work [22]. Slowing cycling by decreasing the laser power and using passive air cooling rather than fans could potentially increase uniformity and efficiency by allowing primer–target interactions to reach equilibrium and by giving PCR enzymes more time for extension, but as this was a rapid PCR study and the system had a similar efficiency to traditional PCR, cycling more slowly was not explored. Moreover, using L-DNA within the reaction volume ensures that annealing and melting switch points are decided by the hybridization state throughout the sample. While DNA hybridization may vary from place to place within the volume, the control algorithm uses the aggregate fluorescence signal for an estimate of the optimal switch points.

Two important aspects of the new system architecture that enhanced both laser systems and enabled faster cycling were increased sampling rate, which reduced variation in the heating and cooling cycle times, and a stable internal instrument temperature. Beyond replacing the LabVIEW software with an open-source version, the C++ hardware management system increased the fluorescence sampling frequency from 3 Hz to 30 Hz. This 10-fold increase in data points enabled the control algorithm to react to the much faster cycling times from the laser heat control system. The cycling control algorithm for the 1370 nm system returned heating and cooling time standard deviations across the baseline of 0.20 and 0.19 s, respectively, over the 480 cycles examined (Figure S4A). The 808 nm fluorescence system had more variation, with standard deviations from the baseline of 0.45 and 0.56 s, respectively, double that of the 1370 nm system (Figure S4B). The laser body itself only reached 22.8 °C, only about 1 °C higher than the ambient temperature of 21.5 °C (Figure S1). This temperature rise is not high enough to make an appreciable difference in cooling time for the instrument or to change its internal temperature significantly. While reaction temperatures are not measured during L-DNA-controlled PCR, the fluorescence values for the switch points—which reflect temperature indirectly—and overall heating and cooling times are stable (Figures S3 and S4), which suggests that the unknown temperatures are also consistent.

These advancements in instrument architecture also suggest that further improvements to L-DNA-controlled PCR are possible with a 1370 nm heating method. In the current version of the algorithm, each heating and cooling switch point is determined during each of the 40 cycles. The low cycling time variation suggests that cycle times can be determined by the baseline portion of the reaction. In addition, since the internal temperature rise is so small, these cycling times do not change throughout the reaction, unlike the previous forced air design, which gradually increased the background temperature. The 1370 nm laser heating does not add to the background temperature. The combination of increased stability and lack of change in the background temperature implies that active switch point calculation is unnecessary once cycling times have been determined. This frees the L-DNA monitoring channels to query additional PCR fluorescence signals that rise after the baseline portion. This increased stability also increases the viability of other potential applications, including product melt temperature analysis.

In summary, both photothermal PCR systems demonstrated that sub-15 min PCR with the adaptive PCR control algorithm is possible and produces results comparable to traditional PCR in a split-sample study. The new system architecture, based on off-the-shelf parts and open-source software, consistently produced similar outcomes to the commercial instrument. However, the optical interference of the nanorods required for 808 nm plasmonic cycling inhibited the control algorithm and PCR probe signals at higher nanorod concentrations, suggesting that the direct infrared heating method is a better option for optically controlled PCR. In a broader context, the 1370 nm system establishes 1370 nm infrared light as a viable heating mechanism for rapid PCR with standard reaction volumes and tubes. Together, the new control system and 1370 nm system are an important step toward a field-deployable adaptive PCR system that can produce rapid results at the point of care.

## 4. Conclusions

Combining 1370 nm photothermal PCR heating with L-DNA hybridization-based control of PCR cycling provides a reliable method of achieving 15 min assay times.

## Figures and Tables

**Table 1:** Oligonucleotide sequences.

Description/Name		Sequence (5′-3′)
Control L-DNA	Anneal Sensor Sequences	TEX—ACTGGGTTTTACAAACCTGTGA
		TCACAGGTTTGTAAAACCCAGTTCCAT—BHQ2
	Melt Sensor Sequences	HEX—ACAAGAAAGGGATCTTCACTCGCGACCGCAAACCGA AGTCGGCGGCTTTTCTGCTGCAAAAACGCTGGACTGGCATG
		CATGCCAGTCCAGCGTTTTTGCAGCAGAAAAGCCGCCGAC TTCGGTTTGCGGTCGCGAGTGAAGATCCCTTTCTTGT—BHQ2
*S. mitis* PCR Assay	Forward Primer	GCCATTGAAGCGGTTACTTTG
	Reverse Primer	CATCCGACATTAACGCAAGTTC
	Probe	FAM—ATGATTGAG—ZEN—CGTGGAACGGTGGGT—IABkFQ
	Synthetic DNA Target	GCCATTGAAGCCGTTACTTTGAACGCAAAAGTGGCTATGATT GAGCGTGGAACGGTGGGTGGAACTTGCGTTAATGTCGGATG

HEX = hexachloro-fluorescein, FAM = fluorescein, TEX = Texas Red, BHQ2 = Black Hole Quencher 2, IABkFQ = Iowa Black FQ, ZEN = Zen internal quencher.

## Data Availability

The data in this study and the instrumentation code are openly available on GitHub at https://doi.org/10.5281/zenodo.14991220, accessed on 7 March 2025.

## Supplementary Materials

The following supporting information can be downloaded at: https://www.mdpi.com/article/10.3390/bios15040258/s1, Figure S1: Temperature of the 1370 nm laser heatsink across a typical 40 cycle sample run remains stable. The temperature slowly rises but eventually tapers off, only reaching 22.8 °C, unlikely to make an appreciable difference in the overall box temperature of 20.8 °C. The data was collected by attaching a K-type thermocouple directly to the heatsink and performing a full 40 cycle PCR run. Figure S2: Alignment of the 1370 nm laser and sample volume are shown via FLIR camera. The sample volume itself is central (outlined in green), while the custom laser heatsink is in the foreground (blue square). The shadow of the sample volume is visible on the power meter in the background (red square) and shows that the laser spot size encompasses the sample volume while not having excessive power loss around the edges. Figure S3: The fluorescence measured for the 1370 nm switch points is more consistent than that measured at the 808 nm switch points, showing that the control algorithm is more reliable without the nanorod optical interference. (A) Deviation from the average fluorescence measured for the melt switch points (red) and anneal switch points (blue) shows the overall consistency of the cycling algorithm for the baselines of all samples performed for 1370 nm standard curve (n = 32, 15 values per sample). The melt points exhibit a wider deviation than the anneal points (std = 10.0 mV vs. 3.71 mV), likely due to the much higher amplitude of the signal for the melted DNA being measured at the high temperature point (avg of 1780 mV vs. 311 mV). (B) Equivalent data for the 808 nm system (n = 31, 15 values per sample), which has a wider distribution in both anneal (blue) and melt (red) switch points (std = 52.4 mV for cooling and 40.02 mV for heating). 5.6% of the reported values for annealing and 23.2% of the reported values for melting are not in the displayed deviation range and are not shown to maintain legibility of the histogram while keeping the same bin sizes as the 1370 nm system for comparison, which further demonstrates the inconsistency of the 808 nm system. Figure S4: As with fluorescence, the heating and cooling times measured on the 1370 nm system are more consistent than those on the 808 nm system. (**A**) Deviation from the average length of heating (red) and cooling (blue) cycles shows the overall consistency of the cycling algorithm for the baselines of all samples performed for the 1370 nm standard curve (n = 32, 15 values per sample). Here, the variation in the heating and cooling times are almost the same (std = 0.19 s vs. 0.20 s), supporting the idea that the strength of the signal exacerbates the apparent deviation in the fluorescence measurements. (**B**) Equivalent data for the 808 nm system (n = 31, 15 values per sample). The 808 nm system also has a wider distribution in terms of time, with larger deviations from the average values in both anneal (blue) and melt (red) times (std = 0.56 and 0.45 respectively), exhibiting the greater inconsistency displayed with the 808 nm system. Outliers at 2.1 and 10.7 seconds are not displayed for the cooling data, and outliers of 2.5, 2.7, 3.1, and 7.2 seconds are not displayed for the heating data to maintain legibility of the histogram while keeping the same bin sizes as the 1370 nm system for comparison. Figure S5: Due to differences in absorption, more of the 808 nm light is absorbed in the reaction volume than the 1370 nm light, but the 1370 nm light heats more evenly in the reaction volume. A simulation of the laser light passing through the sample volume from the left for the (A) 1370 nm laser and (B) the 808 nm laser with a 6.3 nM nanorod concentration shows the percentage of laser intensity that remains at a given depth. The laser intensity decreases exponentially according to Beer’s Law as it penetrates the sample, with the 808 nm absorbed more strongly than the 1370 nm laser. While this has the advantage of more of the power being absorbed within the sample (99% vs. 83% at the thickest part), the distribution of heating—which is proportional to the negative derivative of the intensity—is more uniform in the (C) 1370 nm laser system than the (D) 808 nm laser system. Both heating models assume the laser is a uniform 0.1 W/mm^2^ wavefront. This simple model does not account for convection or conduction, which would cause the heating to be more uniform in both cases.

## References

[1] Obradovic J., Jurisic V., Tosic N., Mrdjanovic J., Perin B., Pavlovic S., Djordjevic N. (2013). Optimization of PCR Conditions for Amplification of GC-Rich EGFR Promoter Sequence. J. Clin. Lab. Anal..

[2] Roux K.H. (2009). Optimization and Troubleshooting in PCR. Cold Spring Harb. Protoc..

[3] Schildkraut C., Lifson S. (1965). Dependence of the melting temperature of DNA on salt concentration. Biopolymers.

[4] Baldini G., Fu-Hua H., Varani G., Cordone L., Fornili S.L., Onori G. (1985). DNA Melting Induced by Alcohols: Role of the Solvent Properties. Il Nuovo C. D.

[5] Anderson D.J., Reischer R.J., Taylor A.J., Wechter W.J. (1984). Preparation and Characterization of Oligonucleotides of D- and L-2’ Deoxyuridine. Nucleosides Nucleotides.

[6] Urata H., Shinohara K. (1991). Mirror Image DNA. J. Am. Chem. Soc..

[7] Adams N.M., Gabella W.E., Hardcastle A.N., Haselton F.R. (2017). Adaptive PCR Based on Hybridization Sensing of Mirror-Image l-DNA. Anal. Chem..

[8] Leelawong M., Adams N.M., Gabella W.E., Wright D.W., Haselton F.R. (2019). Detection of Single-Nucleotide Polymorphism Markers of Antimalarial Drug Resistance Directly from Whole Blood. J. Mol. Diagn..

[9] Spurlock N., Gabella W.E., Nelson D.J., Evans D.T., Pask M.E., Schmitz J.E., Haselton F.R. (2024). Implementing L-DNA Analogs as Mirrors of PCR Reactant Hybridization State: Theoretical and Practical Guidelines for PCR Cycle Control. Anal. Methods.

[10] Euliano E.M., Hardcastle A.N., Victoriano C.M., Gabella W.E., Haselton F.R., Adams N.M. (2019). Multiplexed Adaptive RT-PCR Based on L-DNA Hybridization Monitoring for the Detection of Zika, Dengue, and Chikungunya RNA. Sci. Rep..

[11] Guglielmi G. (2020). Fast Coronavirus Tests: What They Can and Can’t Do. Nature.

[12] Jafek A.R., Harbertson S., Brady H., Samuel R., Gale B.K. (2018). Instrumentation for xPCR Incorporating qPCR and HRMA. Anal. Chem..

[13] Li Z., Ju R., Sekine S., Zhang D., Zhuang S., Yamaguchi Y. (2019). All-in-One Microfluidic Device for on-Site Diagnosis of Pathogens Based on an Integrated Continuous Flow PCR and Electrophoresis Biochip. Lab Chip.

[14] Ouyang Y., Duarte G.R.M., Poe B.L., Riehl P.S., dos Santos F.M., Martin-Didonet C.C.G., Carrilho E., Landers J.P. (2015). A Disposable Laser Print-Cut-Laminate Polyester Microchip for Multiplexed PCR via Infra-Red-Mediated Thermal Control. Anal. Chim. Acta.

[15] Dong X., Liu L., Tu Y., Zhang J., Miao G., Zhang L., Ge S., Xia N., Yu D., Qiu X. (2021). Rapid PCR Powered by Microfluidics: A Quick Review Under the Background of COVID-19 Pandemic. TrAC Trends Anal. Chem..

[16] Terazono H., Hattori A., Takei H., Takeda K., Yasuda K. (2008). Development of 1480 Nm Photothermal High-Speed Real-Time Polymerase Chain Reaction System for Rapid Nucleotide Recognition. Jpn. J. Appl. Phys..

[17] Neuzil P., Zhang C., Pipper J., Oh S., Zhuo L. (2006). Ultra Fast Miniaturized Real-Time PCR: 40 Cycles in Less than Six Minutes. Nucleic Acids Res..

[18] Roche P.J.R., Najih M., Lee S.S., Beitel L.K., Carnevale M.L., Paliouras M., Kirk A.G., Trifiro M.A. (2017). Real Time Plasmonic qPCR: How Fast Is Ultra-Fast? 30 Cycles in 54 Seconds. Analyst.

[19] Nouwairi R.L., Cunha L.L., Turiello R., Scott O., Hickey J., Thomson S., Knowles S., Chapman J.D., Landers J.P. (2022). Ultra-Rapid Real-Time Microfluidic RT-PCR Instrument for Nucleic Acid Analysis. Lab Chip.

[20] Kim H., Vishniakou S., Faris G.W. (2009). Petri Dish PCR: Laser-Heated Reactions in Nanoliter Droplet Arrays. Lab Chip.

[21] Wheeler E.K., Hara C.A., Frank J., Deotte J., Hall S.B., Benett W., Spadaccini C., Beer N.R. (2011). Under-Three Minute PCR: Probing the Limits of Fast Amplification. Analyst.

[22] Mohammadyousef P., Paliouras M., Trifiro M.A., Kirk A.G. (2021). Plasmonic and Label-Free Real-Time Quantitative PCR for Point-of-Care Diagnostics. Analyst.

[23] Evans D.T., Nelson D.J., Pask M.E., Haselton F.R. (2023). A Safer Framework to Evaluate Characterization Technologies of Exhaled Biologic Materials Using Electrospun Nanofibers. Nanoscale.

[24] Kibbe W.A. (2007). OligoCalc: An Online Oligonucleotide Properties Calculator. Nucleic Acids Res..

[25] Uchehara G., Kirk A.G., Trifiro M., Paliouras M., Mohammadyousef P. Real Time Label-Free Monitoring of Plasmonic Polymerase Chain Reaction Products. Proceedings of the SPIE Smart Structures + Nondestructive Evaluation.

[26] Ramakers C., Ruijter J.M., Deprez R.H.L., Moorman A.F.M. (2003). Assumption-Free Analysis of Quantitative Real-Time Polymerase Chain Reaction (PCR) Data. Neurosci. Lett..

[27] Li B., Ellington A.D., Chen X. (2011). Rational, Modular Adaptation of Enzyme-Free DNA Circuits to Multiple Detection Methods. Nucleic Acids Res..

