# Supplementary material for: Achievement of 15-Minute Adaptive PCR Benchmark with 1370 nm Laser Heating
Source: Biosensors (Basel). 2025 Apr 17;15(4):258. doi: 10.3390/bios15040258 (PMC12026111; doi:10.3390/bios15040258)
Supplement: Supplementary file 1 [file biosensors-15-00258-s001.zip › biosensors-3545306-supplementary.pdf]

Supplemental Information

Achievement of 15-Minute Adaptive PCR Benchmark with 1370 nm Laser Heating

Nicholas Spurlock [1], Rosana Alfaro [1], William E. Gabella [2], Kunal Chugh [1], Megan E. Pask [1], Franz Baudenbacher [1] and Frederick R. Haselton [1]

This supplemental data is divided into three sections. The first and second sections provide additional measurements in support of the 1370 nm system's hardware performance. The first section illustrates the thermal performance and alignment, and the second provides data that supports the performance of the new software architecture and control system. The third section contains a simple simulation of light within the sample volume, and the heat resulting from absorbance.

Thermal behavior and alignment information for the 1370 nm laser instrument

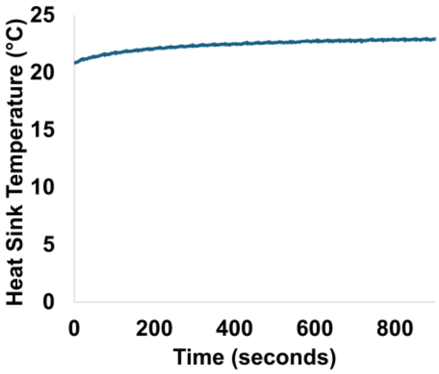

Figure S1: Temperature of the 1370 nm laser heatsink across a typical 40 cycle sample run remains stable. The temperature slowly rises but eventually tapers off, only reaching 22.8 °C, unlikely to make an appreciable difference in the overall box temperature of 20.8 °C. The data was collected by attaching a K-type thermocouple directly to the heatsink and performing a full 40 cycle PCR run.

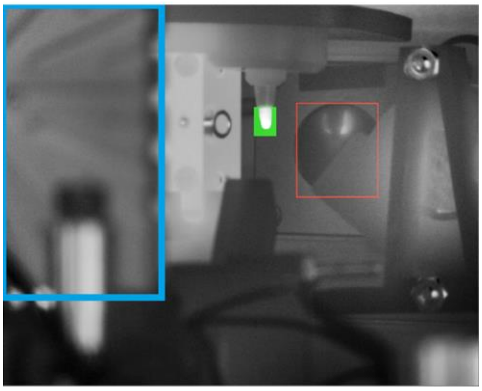

Figure S2: Alignment of the 1370 nm laser and sample volume are shown via FLIR camera. The sample volume itself is central (outlined in green), while the custom laser heatsink is in the foreground (blue square). The shadow of the sample volume is visible on the power meter in the background (red square) and shows that the laser spot size encompasses the sample volume while not having excessive power loss around the edges.

Variation in cycling switch point for the two heating methods

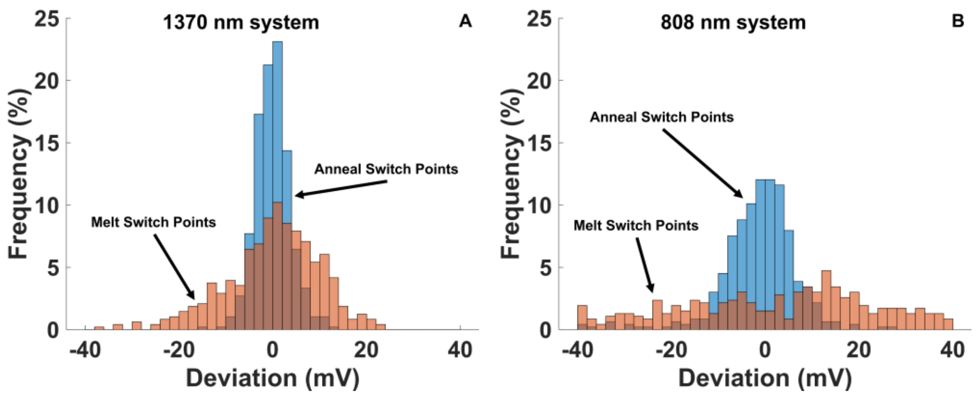

Figure S3: The fluorescence measured for the 1370 nm switch points is more consistent than that measured at the 808 nm switch points, showing that the control algorithm is more reliable without the nanorod optical interference. **(a)** Deviation from the average fluorescence measured for the melt switch points (red) and anneal switch points (blue) shows the overall consistency of the cycling algorithm for the baselines of all samples performed for 1370 nm standard curve (n= 32, 15 values per sample). The melt points exhibit a wider deviation than the anneal points (std = 10.0 mV vs 3.71 mV), likely due to the much higher amplitude of the signal for the melted DNA being measured at the high temperature point (avg of 1780 mV vs 311 mV). **(b)** Equivalent data for the 808 nm system (n = 31, 15 values per sample), which has a wider distribution in both anneal (blue) and melt (red) switch points (std = 52.4 mV for cooling and 40.02 mV for heating). 5.6% of the reported values for annealing and 23.2% of the reported values for melting are not in the displayed deviation range and are not shown to maintain legibility of the histogram while keeping the same bin sizes as the 1370 nm system for comparison, which further demonstrates the inconsistency of the 808 nm system.

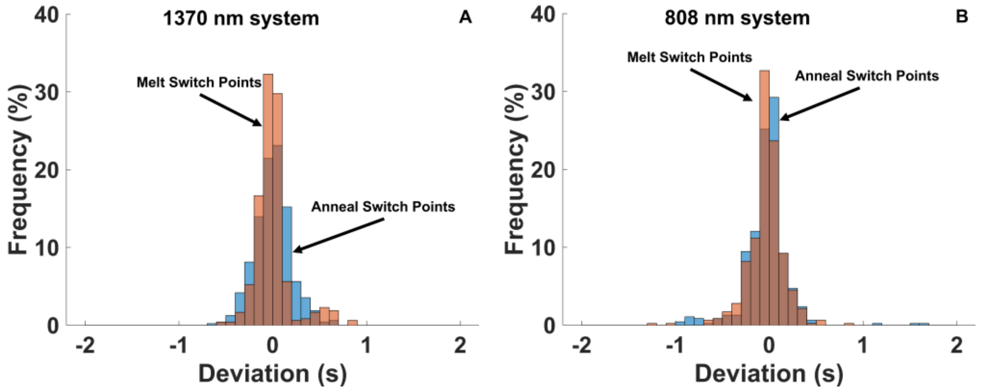

Figure S4: As with fluorescence, the heating and cooling times measured on the 1370 nm system are more consistent than those on the 808 nm system. **(a)** Deviation from the average length of heating (red) and cooling (blue) cycles shows the overall consistency of the cycling algorithm for the baselines of all samples performed for the 1370 nm standard curve (n=32, 15 values per sample). Here, the variation in the heating and cooling times are almost the same (std = 0.19s vs 0.20s), supporting the idea that the strength of the signal exacerbates the apparent deviation in the fluorescence measurements. **(b)** Equivalent data for the 808 nm system (n=31, 15 values per sample). The 808 nm system also has a wider distribution in terms of time, with larger deviations from the average values in both anneal (blue) and melt (red) times (std = 0.56 and 0.45 respectively), exhibiting the greater inconsistency displayed with the 808 nm system. Outliers at 2.1 and 10.7 seconds are not displayed for the cooling data, and outliers of 2.5, 2.7, 3.1, and 7.2 seconds are not displayed for the heating data to maintain legibility of the histogram while keeping the same bin sizes as the 1370 nm system for comparison.

Laser light and theoretical heat distribution for both heating methods.

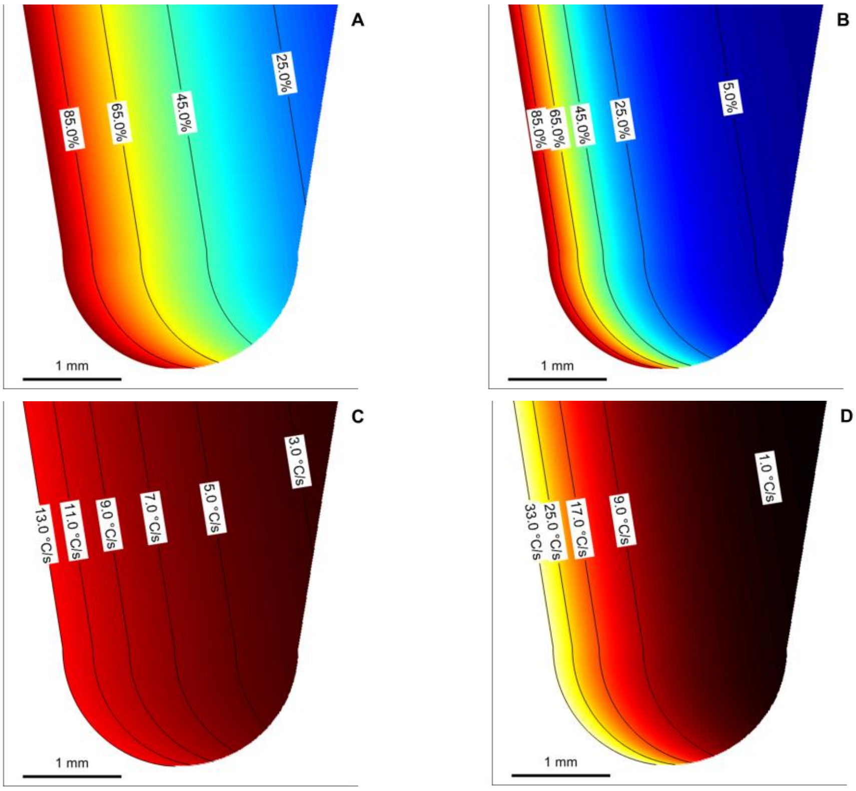

Figure S5: Due to differences in absorption, more of the 808 nm light is absorbed in the reaction volume than the 1370 nm light, but the 1370 nm light heats more evenly in the reaction volume. A simulation of the laser light passing through the sample volume from the left for the (a) 1370 nm laser and (b) the 808 nm laser with a 6.3 nM nanorod concentration shows the percentage of laser intensity that remains at a given depth. The laser intensity decreases exponentially according to Beer's Law as it penetrates the sample, with the 808 nm absorbed more strongly than the 1370 nm laser. While this has the advantage of more of the power being absorbed within the sample (99% versus 83% at the thickest part), the distribution of heating—which is proportional to the negative derivative of the intensity—is more uniform in the (c) 1370 nm laser system than the (d) 808 nm laser system. Both heating models assume the laser is a uniform 0.1 W/mm^2 wavefront. This simple model does not account for convection or conduction, which would cause the heating to be more uniform in both cases.
